# Diversity of the midstream urine microbiome in adults with chronic kidney disease

**DOI:** 10.1007/s11255-018-1860-7

**Published:** 2018-04-12

**Authors:** Holly Kramer, Gina Kuffel, Krystal Thomas-White, Alan J. Wolfe, Kavitha Vellanki, David J. Leehey, Vinod K. Bansal, Linda Brubaker, Robert Flanigan, Julia Koval, Anuradha Wadhwa, Michael J. Zilliox

**Affiliations:** 1 Department of Public Health Sciences, Loyola University Chicago, 2160 S. First Avenue, Maywood, IL 60153 USA; 2 Medicine, Division of Nephrology and Hypertension, Loyola University Chicago, Maywood, IL USA; 3 Departments of Microbiology and Immunology, Loyola University Chicago, Maywood, IL USA; 4 Obstetrics and Gynecology, Loyola University Chicago, Maywood, IL USA; 5 Urology, Stritch School of Medicine, Loyola University Chicago, Maywood, IL USA; 6 Hines VA Medical Center, Hines, IL USA; 7 Present Address: Evy Health, 325 Sharon Park Dr., Suite 522, Menlo Park, CA USA; 8 Department of Reproductive Medicine, Division of Female Pelvic Medicine and Reconstructive Surgery, University of California San Diego, La Jolla, CA USA

**Keywords:** Urinary microbiome, Chronic kidney disease, Microbiome diversity, Urinary symptoms

## Abstract

**Purpose:**

To examine the characteristics of the midstream urine microbiome in adults with stage 3–5 non-dialysis-dependent chronic kidney disease (CKD).

**Methods:**

Patients with non-dialysis-dependent CKD (estimated glomerular filtration rate [eGFR] < 60 ml/min/1.73 m^2^) and diuretic use were recruited from outpatient nephrology clinics. Midstream voided urine specimens were collected using the clean-catch method. The bacterial composition was determined by sequencing the hypervariable (V4) region of the bacterial 16S ribosomal RNA gene. Extraction negative controls (no urine) were included to assess the contribution of extraneous DNA from possible sources of contamination. Midstream urine microbiome diversity was assessed with the inverse Simpson, Chao and Shannon indices. The diversity measures were further examined by demographic characteristics and by comorbidities.

**Results:**

The cohort of 41 women and 36 men with detectable bacterial DNA in their urine samples had a mean age of 71.5 years (standard deviation [SD] 7.9) years (range 60–91 years). The majority were white (68.0%) and a substantial minority were African-American (29.3%) The mean eGFR was 27.2 (SD 13.6) ml/min/1.73 m^2^. Most men (72.2%) were circumcised and 16.6% reported a remote history of prostate cancer. Many midstream voided urine specimens were dominated (> 50% reads) by the genera *Corynebacterium* (*n* = 11), *Staphylococcus* (*n* = 9), *Streptococcus* (*n* = 7), *Lactobacillus* (*n* = 7), *Gardnerella* (*n* = 7), *Prevotella* (*n* = 4), *Escherichia_Shigella* (*n* = 3), and *Enterobacteriaceae* (*n* = 2); the rest lacked a dominant genus. The samples had high levels of diversity, as measured by the inverse Simpson [7.24 (95% CI 6.76, 7.81)], Chao [558.24 (95% CI 381.70, 879.35)], and Shannon indices [2.60 (95% CI 2.51, 2.69)]. Diversity measures were generally higher in participants with urgency urinary incontinence and higher estimated glomerular filtration rate (eGFR). After controlling for demographics and diabetes status, microbiome diversity was significantly associated with estimated eGFR (*P* < 0.05).

**Conclusions:**

The midstream voided urine microbiome of older adults with stage 3–5 non-dialysis-dependent CKD is diverse. Greater microbiome diversity is associated with higher eGFR.

## Introduction

Bacterial communities in the bladders of men and women without clinical urinary tract infections (UTI) have been discovered and emerging research suggests that the urinary microbiome may influence bladder health [1–6]. Although the vast majority of genitourinary bacteria, which includes bacteria from the urethra, vagina, vulva, and bladder, cannot be cultured by standard clinical microbiology urine culture methods [7, 8], they can be identified using high throughput 16S rRNA sequencing or new enhanced urine culture techniques [2, 4, 8–10]. The presence of urinary bacteria does not indicate UTI, as the bacteria that comprise the resident urinary microbiome differ from those associated with clinical UTIs [5, 6, 11, 12]. While few studies have examined the diversity of the urine microbiome, current evidence links microbial diversity in catheterized urine specimens with urgency urinary incontinence (UUI) and response to treatment for lower urinary tract symptoms, especially those associated with UUI [13, 14]. Midstream voided urine microbiome diversity also has been associated with lower urinary symptoms, as well as with hormonal status and body mass index [15]. These preliminary findings are consistent with other non-urine microbiome studies showing that human microbiome diversity may influence health status. For example, low gut microbiome diversity is associated with inflammatory bowel disease [16, 17], while high vaginal microbiome diversity is associated with bacterial vaginosis [18].

Chronic kidney disease (CKD) is a disease generally associated with advanced age and comorbidities, such as diabetes and obesity. The older age of adults with CKD along with accompanying comorbidities may influence midstream urine microbiome diversity and could influence urinary symptoms and bladder health. Diabetes leads to high ambient urinary glucose levels, which may increase bacterial growth and influence urinary microbiome diversity. Severe reductions in estimated glomerular filtration rate (eGFR) also may influence bacterial growth via its effects on production of uromodulin, produced exclusively by the renal tubules [19–22] and promotes urinary excretion of bacteria [23]. Urinary symptoms are troublesome for older adults with CKD, and we have previously demonstrated a high burden of urinary symptoms among older adults with CKD receiving diuretics [24].

This single-center pilot study examined the presence and diversity of the midstream voided urinary microbiome among older adults with CKD who require diuretics for disease management. It further examined the association of the observed microbiome diversity with eGFR, comorbidities and urinary symptoms. We hypothesized that the midstream urine microbiome in adults with CKD in the absence of a clinical UTI would be diverse and that this diversity would be associated with eGFR, comorbidities, and urinary symptoms.

## Methods

### Study population

Details of the study have been previously published [24]. The Loyola University Chicago Health Sciences Division Institutional Review Board approved the study, and all participants provided written informed consent. Participants were recruited from Loyola Outpatient Center Nephrology clinics during a routine outpatient clinic visit from November 1, 2014, to June 30, 2015. Adults were invited to participate if they were aged ≥ 60 years with an eGFR < 60 ml/min/1.73 m^2^ based on the Modification of Diet in Renal Disease [25] formula within four weeks of study enrollment and were taking diuretics (thiazide or loop diuretics, potassium sparing diuretics, or aldosterone blockade medications) either solely or in addition to other medications for hypertension management. Diuretic use was an inclusion criterion because the study was specifically designed to examine urinary symptoms in adults with CKD receiving diuretics. Patients were excluded from the study if they were receiving any immunosuppression medications, had a history of a neobladder or ileal conduit, had used antibiotics within the past 4 weeks or had any active cancer treatment or urinary instrumentation within the past 6 months. A total of 135 patient participants were asked to enroll in the study, 103 provided consent, and 99 contributed completed urinary symptom questionnaires. One participant withdrew consent from the study after completing the questionnaires, and five participants did not provide a urine sample. An additional 11 samples were contaminated, and 5 had too few sequence reads. Thus, the analyzed cohort with detectable urine DNA included 77 participants (41 females and 36 males).

### Midstream urinary microbiome

Midstream urines were obtained by the clean-catch method with a special emphasis on proper clinical collection procedures. The urine specimens were collected in 60 ml sterile urine cups containing AssayAssure (Sierra Molecular, Incline Village, NV), a preservative that maintains bacteriostasis by retarding the reproduction and lysis of bacteria. The preservative also minimizes freeze/thaw damage to nucleic acids and acts like a chemical refrigerator by preserving specimens without refrigeration or freezing from 7 to 45 days.

### Demographic variables, medication use and medical history

All participants completed self-administered questionnaires that queried cancer history (including prostate), and previous surgeries (including prostatectomy or hysterectomy), circumcision status (men only). Medication use, diabetes status (physician diagnosis and/or use of glucose lowering medication), body mass index (BMI) and history of prostate cancer and benign prostatic hypertrophy were obtained from the electronic medical record. Stage of CKD was defined as stage 3 if eGFR was between 59 and 30 ml/min/1.73 m^2^, stage 4 if eGFR was between 15 and 29 ml/min/1.73 m^2^ and stage 5 if eGFR was < 15 ml/min/1.73 m^2^ [26]. Presence of urgency urinary incontinence (UUI) and nocturia were assessed using the National Health and Nutrition Examination Surveys (NHANES) 2013 kidney condition items that assesses urinary leakage, amount, and frequency over the current month and over the previous 12 months [27]. Presence of UUI was defined as responding “Yes” to the question “During the past 12 months have you leaked or lost control of even a small amount of urine with an urge or pressure to urinate and you couldn’t get to the toilet fast enough?” Nocturia was defined as reporting awakening 2 or more times per night to urinate.

### DNA extraction

Genomic DNA was extracted from midstream voided urine samples using a validated mixture of lysozyme and mutanolysin [28] and the DNeasy Blood and Tissue Kit (Qiagen, Valencia, CA). All steps were performed in a UV irradiated, HEPA-filtered PCR workstation to minimize potential contaminants. The amount of DNA in each sample was quantified using the Qubit 2.0 Flurometer (Life Technologies, Carlsbad, CA). The microbial composition was determined by sequencing the hypervariable (V4) region of the bacterial 16S ribosomal RNA gene, as previously described [2, 14]. The V4 region is about 250 base-pairs long and can be used to classify most bacteria to the genus level. Sequencing-ready libraries were generated using a 2-step polymerase chain reaction (PCR). The V4 region was amplified using modified universal primers 515F and 806R. In a limited cycle PCR, Illumina sequencing adapters and dual-index barcodes were added to the amplified targets. Extraction negative controls (no urine) were included to assess the contribution of extraneous DNA from possible sources of contamination. The final PCR products were purified using the Agencourt AMPure XP system (Beckman Coulter, Brea, CA) and pooled together in equimolar concentrations to create a final library of barcoded fragments ready for sequencing. The samples were sequenced on the Illumina MiSeq bench-top sequencer rendering 250 base-pair paired-end reads (Illumina, San Diego, CA). Sequencing data was deposited in SRA accession SRP127363.

### Data analysis

Raw sequencing reads were processed using the Mothur software package (v. 1.31.2) [29] to remove low quality and chimeric sequences. The ribosomal database project classifier (RDP) [30] was used to generate taxonomic classifications of the sequencing reads from phylum to genus level. Samples were subsampled at a depth of 2000 sequencing reads; five samples were eliminated from downstream analysis due to not meeting this threshold. The taxonomic information was then used to split the sequences into bins using a 97% similarity threshold to assign operational taxonomic units (OTUs), using the clustering algorithm built into the Mothur software; this resulted in 8658 OTUs. The relative abundance of taxonomic classifications was calculated at the genus level for each sample using the R package Phyloseq (v. 1.19.1). Taxa that did not constitute 1% of the total sequencing reads and/or greater than 40% of a single sample’s sequencing reads comprise the taxa category “Other.” Samples were clustered using only the identified taxa to assess variance using distance matrices and the Bray–Curtis index in the R package vegan (v. 2.4.3), producing a dendrogram in which the short branches link similar samples and longer branches link more dissimilar samples; this segregates the dendrogram into distinct groups or clades. Unclassified sequencing reads and reads that were grouped into the “Other” category were not used for clustering. The relative abundance graph was then aligned to the dendrogram to delineate the clades of the tree by the predominant organism, termed “urotype,” above a defined threshold. We also plotted the proportion of sequences per genus across all patient samples to help visualize the total number of genera represented in the midstream voided urine samples. Alpha diversity was assessed using three types of measurements. The total number of unique taxa was estimated by the CHAO measure, and the species richness was measured by the inverse of the Simpson index. The Shannon index was used to assess both the richness and evenness or equality of representation of taxa within an environment. Higher values of these indices indicate higher diversity.

### Statistical analysis

Descriptive statistics of the 77 study participants were examined by sex. The midstream urine microbiome diversity measures were examined by demographic characteristics and by comorbidities (diabetes, obesity, UUI and CKD stage) in the total sample. Due to a non-normal distribution, the diversity measures were log-transformed for analyses. To determine significance of differences in diversity measures, the unpaired *t* test was used to compare log-transformed values by a given characteristic and geometric mean values were reported. The ANOVA test was used to examine significant differences in log-transformed diversity measures across CKD stages 3, 4 and 5. If the overall test was significant (*P* < 0.05), then log-transformed diversity measures for CKD stages 4 and 5 were compared to CKD stage 3 using an unpaired *t* test. Linear regression was used to examine the adjusted association of demographic characteristics and comorbidities with log-transformed diversity measures as the dependent variable. Models include age, BMI and eGFR as continuous variables and male sex, diabetes, and white race as dichotomous variables. Analyses were completed using STATA v13.

## Results

The cohort of 41 women and 36 men with detectable bacterial DNA in their midstream voided urine sample had a mean age of 71.5 years (standard deviation [SD] 7.9) (range 60–91 years); race/ethnicity was reported as white (68.0%), African-American (29.3%) or other race/ethnicity (2.7%). Mean eGFR was 27.2 (SD 13.5) ml/min/1.73 m^2^; nocturia and UUI were reported by 63.6% and 46.8%, respectively. Table 1 shows participant characteristics by sex. More than half (69.4% of men and 70.0% of women) had diabetes. Urinary symptoms were common in men and women. Nocturia was reported by 77.8 and 51.2% of men and women, respectively, whereas UUI was reported by 41.7 and 51.2% of men and women, respectively. Most men (72.2%) were circumcised and 16.6% reported a remote history of prostate cancer.

**Table 1:** Characteristics of the study participants

	Male (*n* = 36)	Female (*n* = 41)
Age (years)	76.6 (7.6)	70.5 (8.2)
Race		
White	69.4%	63.4%
Black	25.0%	31.7%
Other race	5.6%	4.9%
Hispanic ethnicity	7.7%	2.4%
BMI (kg/m^2^)	30.8 (6.9)	32.0 (5.9)
Obesity	50.0%	58.5%
Diabetes	69.4%	70.0%
eGFR (ml/min/1.73 m^2^)	29.5 (6.2)	27.5 (13.5)
Stage 3 CKD	44.4%	41.5%
Stage 4 CKD	27.8%	36.0%
Stage 5 CKD	27.8%	21.9%
Urgency-UI	41.7%	51.2%
Nocturia^a^	77.8%	51.2%
Circumcised	72.2%	–
Prostate cancer history	16.6%	–
Benign prostatic hyperplasia	30.6%	–

*UI* urinary incontinence, *eGFR* estimated glomerular filtration rate, *CKD* chronic kidney disease; stage 3, 4 and 5 defined as eGFR 59–30, 29–15 and < 15 ml/min/1.73 m^2^ but not on dialysis, respectively

^a^Nocturia defined as waking up at least twice per night to urinate

**Fig. 1:**
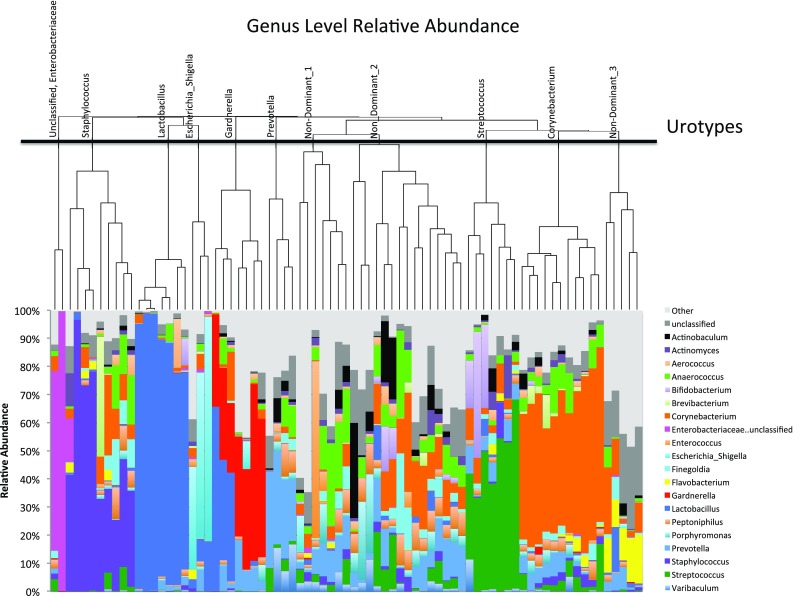
Genus level relative abundance. Each vertical bar represents the midstream urine of a study participant with percent of total classified reads to the genus level on the *y*-axis. The Bray–Curtis dissimilarity index is calculated to generate the dendrogram and the thick black line shows the urotype cut-off

Figure 1 shows bacterial profiles in terms of relative abundance, clustered by Bray–Curtis dissimilarity and depicted as a dendrogram. Many samples were dominated (> 50% reads) by a singe highly prevalent taxon, most often the genus *Corynebacterium* (*n* = 11), followed by the genera *Staphylococcus* (*n* = 9), *Lactobacillus* (*n* = 7), *Gardnerella* (*n* = 7), *Streptococcus* (*n* = 7), *Prevotella* (*n* = 4), and *Esherichia_Shigella* (*n* = 3), as well as the family Enterobacteriaceae (*n* = 2). The remaining 27 samples had no dominant or highly prevalent taxon. In the total sample, the inverse Simpson and Chao1 indices, which measure richness (abundance), were 7.24 (95% CI 6.76, 7.81) and 558.24 (95% CI 381.70, 879.35), respectively. The Shannon index, which accounts for both richness and evenness (distribution) of present species, was 2.60 (95% CI 2.51, 2.69).

**Fig. 2:**
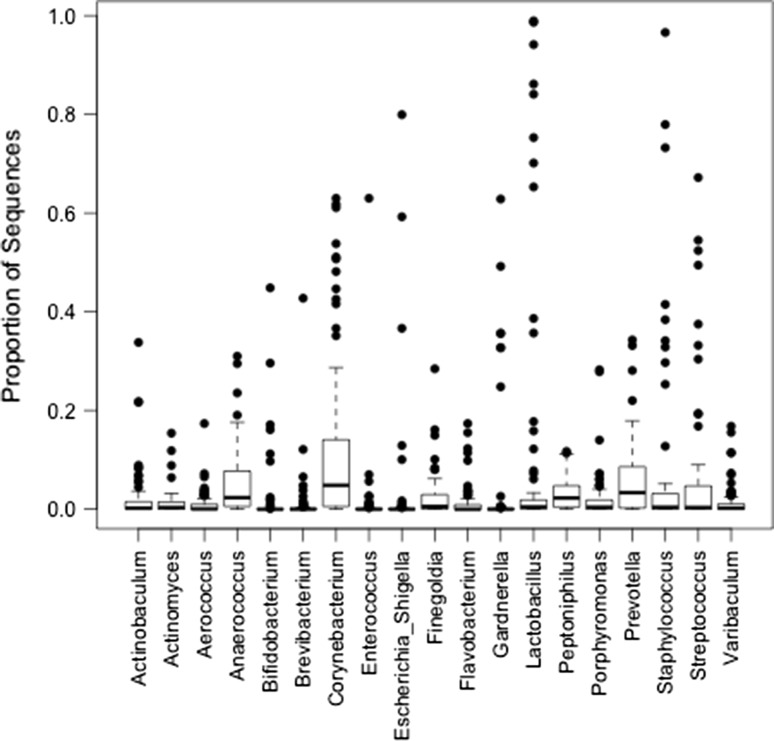
The across sample distribution is shown for the genera found at > 1% abundance in at least one of the 77 samples. Note that very few genera constitute > 50% of the proportion of sequences in a sample, highlighting the microbiome diversity in these patients

This diversity was also observed in the across sample distribution, which plots the proportion of sequences per genus across all 77 patient samples (Fig. 2). The total number of genera represented in the samples was 19, as represented by the *x*-axis. Very few urine samples were overwhelmingly dominated by a single genus. Thus, most urine samples with a dominant urotype (> 50% of sequences) contained several other prominent genera. In most samples, each genus was detected as a low proportion of all sequences. Table 2 shows the midstream urine microbiome diversity measures (Inverse Simpson, CHAO and Shannon indices) by demographic characteristics and comorbidities. No significant difference was noted relative to age, sex, ethnicity or either diabetes or obesity status. Overall, diversity measures were higher among men versus women, but the differences did not meet statistical significance. In contrast, significant differences in diversity measures were observed for those reporting UUI versus those without UUI. In the total study group, diversity measures also differed significantly with respect to CKD stage (Table 3). The highest diversity measures were associated with CKD stage 3, whereas the lowest diversity measures were generally noted with CKD stage 5. After controlling for demographics and diabetes status, only eGFR remained significantly and consistently associated with diversity measures with higher eGFR associated with higher measures of diversity (Table 4).

**Table 2:** Geometric means of diversity measures by patient characteristics

Inverse Simpson		CHAO		Shannon	
Age ≥ 75 years	Age < 75 years	Age ≥ 75 years	Age < 75 years	Age ≥ 75 years	Age < 75 years
7.33 (6.84, 7.88)	7.20 (6.72, 7.77)	555.57 (380.82, 882.42)	558.21 (382.18, 877.70)	2.65 (2.55, 2.74)	2.57 (2.47, 2.67)
Male	Female	Male	Female	Male	Female
7.24 (6.76, 7.81)	6.09 (5.71, 6.52)	595.83 (424.68, 892.45)	527.19 (347.57, 868.0.1)	2.99 (2.90, 3.08)	2.30 (2.21, 2.40)*
White race	Non-white race	White race	Non-white race	White race	Non-white race
8.29 (7.73, 8.93)	5.67 (5.30, 6.11)	552.76 (379.60, 867.19)	560.72 (383.00, 883.74)	2.70 (2.61, 2.80)	2.43 (2.33, 2.52)
Obese	Non-obese	Obese	Non-obese	Obese	Non-obese
6.51 (6.08, 7.00)	8.24 (7.67, 8.90)	571.12 (378.98, 929.71)	543.16 (385.00, 822.51)	2.44 (2.34, 2.53)	2.81 (2.72, 2.90)
Diabetic	Non-diabetic	Diabetic	Non-diabetic	Diabetic	Non-diabetic
7.32 (6.83, 7.89)	7.61 (7.11, 8.20)	555.33 (383.40, 866.03)	560.93 (376.05, 901.96)	2.70 (2.61, 2.80)	2.41 (2.31, 2.51)
UUI+	UUI−	UUI+	UUI−	*UUI+	UUI−
8.40 (7.81, 9.09)	6.36 (5.96, 6.83)	627.97 (420.06, 1009.73)	503.42 (350.92, 778.82)	2.83 (2.74, 2.95)	2.41 (2.31, 2.51)
Nocturia+	Nocturia−	Nocturia+	Nocturia−	Nocturia+	Nocturia−
6.87 (6.39, 7.43)	8.09 (7.59, 8.67)	519.51 (366.83, 791.96)	627.20 (407.41, 1041.23)	2.59 (2.50, 2.69)	2.62 (2.52, 2.71)

*P < 0.05

**Table 3:** Geometric means of diversity measures by chronic kidney disease (CKD) stage

Diversity measure	CKD stage 3 (*n* = 33)	CKD stage 4 (*n* = 25)	CKD stage 5 (*n* = 19)	Overall *P* value
Inverse Simpson index	10.48 (7.54, 14.44)	5.53 (3.86, 8.00)*	5.47 (3.42, 8.67)*	0.01
CHAO	614.00 (533.79, 699.24)	555.57 (464.05, 658.52)*	478.19 (507.76, 561.16)*	0.04
Shannon	2.99 (2.91, 3.08)	2.37 (2.27, 2.46)*	2.31 (2.20, 2.41)	0.4

*P < 0.05 versus CKD stage 3

**Table 4:** Results of multivariable linear regression analyses of log-transformed diversity measures

	Inverse Simpson+		CHAO+		Shannon index	
	Beta (95% CI)	*P* value	Beta (95% CI)	*P* value	Beta (95% CI)	*P* value
Age	0.01 (−0.02, 0.04)	0.62	0.004 (−0.01, 0.02)	0.45	−0.01 (−0.02, 0.01)	0.9
Male	0.40 (0.30, 0.83)	0.07	0.13 (−0.05, 0.31)	0.14	0.28 (0.06, 0.51)	0.01
White race (vs. non-white)	0.35 (−0.12, 0.82)	0.15	−0.01 (−0.20, 0.18)	0.90	0.06 (−0.18, 0.30)	0.62
BMI	0.02 (−0.02, 0.05)	0.4	0.02 (0.001, 0.03)	0.04	0.001 (−0.017, 0.02)	0.88
eGFR	0.02 (0.01, 0.04)	0.008	0.01 (0.001. 0.02)	0.02	0.01 (.0002, 0.02)	0.04
Diabetes status	−0.02 (−0.48, 0.45)	0.94	0.01 (−0.18, 0.20)	0.9	0.01 (−0.11, 0.38)	0.27

## Discussion

This study shows that the midstream voided urine microbiomes of adults with CKD stage 3–5 are diverse. In the total sample and among women, the diversity measures were dramatically higher than those from another study that evaluated midstream voided urine samples obtained by the clean catch method in women with stress urinary incontinence [15]. In that prior study of midstream voided urine samples in women without CKD, they reported an inverse Simpson index of only 1.86 and a Chao index of 124.08, values several fold lower (3.9 and 4.5 fold lower, respectively), than values reported in this study of adults with CKD. However, information on midstream urine microbiome diversity and potential influencing factors remain very limited.

In the current study, we also noted higher diversity measures in the midstream urine microbiome from adults with UUI versus those without UUI. The association of urine microbiome diversity with urinary health measures remains poorly explored. One study analyzed urine obtained by transurethral catheter and found that a more diverse urinary microbiome for women correlates with less robust treatment response for UUI with an anticholinergic treatment [13]. In that study, compared to women with less diverse urine microbiomes, those with more diverse microbiomes either required a higher dose of the anticholinergic for the same response or did not respond to the anticholinergic at all [13]. Another smaller study of 10 women with UUI and 10 women with normal bladder function found that lower urine microbiome diversity from transurethral catheterized specimens was associated with increased UUI severity [31]. Others have also reported lower midstream urine microbiome diversity in women with interstitial cystitis compared to women without urinary symptoms [32, 33]. Additionally, one previous small study of midstream urine specimens from 16 persons age 20–70 + years showed that midstream urine microbiome diversity generally decreased with age with the lowest diversity noted in adults aged ≥ 70 years [34].

Our finding that the midstream urine microbiome diversity measures were generally lower with reduced eGFR, even after adjustment for demographics and diabetes status, shows a potentially intriguing link between eGFR and the midstream urine microbiome diversity. This association will require additional study to determine contributing factors such as alterations in urinary antimicrobial peptides. In the renal tubules, the tacky uromodulin proteins stick to bacteria; this leads to formation of larger particles that are more readily excreted by the kidney [19, 23] and reduces the risk of a UTI [19–22]. It is possible that kidney function decline alters the secretion of uromodulin and other antimicrobial peptides into the urine, which may influence urinary microbiome diversity, but this remains an untested hypothesis. While uromodulin concentrations generally decrease as GFR declines [35], large inter-individual variability in uromodulin urine concentrations exists. Other unmeasured factors, such as vaginal and urethral bacterial growth, may also be influenced by kidney disease and alter the urine microbiome diversity.

Microbial assessments obtained using voided samples may include microbial contributions from adjacent pelvic niches, especially in women. Despite these limitations, the use of voided samples avoids the participant burden of urinary catheterization and the potential for alteration of the urinary microbial community. The microbes detected in this analysis cannot be confirmed to originate from the bladder itself. However, the characteristics of the midstream urine microbiome in women have similarities to urine microbiomes in catheterized urine specimens [4, 14, 15]. Few studies have examined the voided urine microbiome characteristics in men [36, 37].

While these pilot data are hypothesis generating, we cannot determine whether kidney disease influences the midstream urinary microbiome or vice versa. Because participants contributed a single urine sample, we cannot comment on microbial stability over time. Future studies that examine the urine microbiome at several time points can determine intra-individual variability of the urine microbiome and its change over time.

In summary, this study shows that the midstream urine microbiomes are diverse in adults with CKD and that this diversity appears to be lower with more advanced CKD. These findings should be confirmed in other populations with CKD. Future studies should also examine the characteristics of the urinary microbiome using catheterized specimens and examine the association of the urinary microbiome characteristics with lower urinary tract symptoms in adults with CKD.
